# Psychological and sociodemographic factors associated with hypoactive sexual desire in Ecuadorian women

**DOI:** 10.3389/fsoc.2024.1489845

**Published:** 2024-11-25

**Authors:** Doris Pérez-Vega, Andrés Subía-Arellano, Jorge Buele

**Affiliations:** ^1^Carrera de Psicología, Facultad de Ciencias Sociales y Humanas, Universidad Tecnológica Indoamérica, Quito, Ecuador; ^2^Carrera de Ingeniería Industrial, Facultad de Ingenierías, Universidad Tecnológica Indoamérica, Ambato, Ecuador

**Keywords:** inhibited sexual desire, hypoactive sexual desire, erotophilia, erotophobia, women

## Abstract

**Introduction:**

Human sexuality is a multifaceted process, and sexual desire plays a central role in the triphasic model of the sexual response cycle, as proposed by Helen Singer Kaplan.

**Methods:**

In this cross-sectional correlational study, we examined the relationship between various sociodemographic factors, such as age and motherhood, and sexual variables, including erotophobia, erotophilia, homophobia, and unconventional sex, with hypoactive sexual desire in women from Quito, Ecuador. The study sample comprised 421 women between the ages of 18 and 50, who were administered the Revised Sexual Opinion Survey and the Inhibited Sexual Desire Scale to assess their sexual attitudes and levels of desire.

**Results:**

The findings revealed that age (*F* = 7.13, *p* < 0.001) and motherhood (*F* = 13.72, *p* < 0.001) had a significant impact on inhibited sexual desire. Furthermore, significant correlations were observed between inhibited sexual desire and age (*r* = 0.16, *p* < 0.001), motherhood (*r* = 0.18, *p* < 0.001), erotophobia (*r* = 0.19, *p* < 0.001), erotophilia (*r* = −0.21, *p* < 0.001), and homophobia (*r* = −0.18, *p* < 0.001).

**Discussion:**

These results suggest that women who are older, mothers, or have higher levels of erotophobia are more likely to experience hypoactive sexual desire. In contrast, higher levels of erotophilia and homophobia were inversely related to hypoactive sexual desire. This contributes to a deeper understanding of how different personal and sexual attitudes influence sexual desire in Ecuadorian women.

## 1 Introduction

Hypoactive sexual desire in women is a disorder that occurs when the lack of sexual thoughts, fantasies, and sexual activity lasts for a long time (Kingsberg et al., 2019). This problem negatively impacts women’s psychological well-being and quality of life since it prevents them from participating in sexual activities due to sadness, worry, frustration, and other feelings (O’Loughlin and Brotto, 2020). Hypoactive sexual desire disorder (HSDD) affects approximately 8.9% of women aged 18–44, 12.3% aged 45–64, and 7.4% over 65, with distress levels decreasing with age (Parish and Hahn, 2016). Women who suffer from it may feel unhappy and deprived of comfort due to their strong sexual desire and lack of satisfaction or decreased sexual desire (Gore-Gorszewska, 2021).

Hypoactive sexual desire has been incorporated into the fifth edition of the Diagnostic and Statistical Manual of Mental Disorders within a new category, “female sexual interest and arousal disorder” (Kingsberg and Woodard, 2015), in which the disorders have been merged, hypoactive sexual desire and female sexual arousal. The definition of normal sexual desire in women is variable. It depends on various factors, such as previous sexual experiences, culture, biological drive, neuroendocrine changes, and stressors that can influence the perception of sexual desire in each woman (Arnow et al., 2009; Leavitt et al., 2019).

Various hormonal and neurotransmitter factors influence sexual desire in women. Norepinephrine, melanocortin, oxytocin, testosterone, and dopamine play important roles in sexual desire (Kingsberg et al., 2015). Testosterone and estrogen levels are essential for sexual desire, along with high levels of dopamine and norepinephrine, while serotonin acts as a sexual inhibitor (Jayne et al., 2017). Dopamine has a significant role in the modulation of desire. It seems to increase the subjective feeling of arousal and the desire to continue sexual activity when stimulation has begun. On the other hand, androgens improve hypoactive sexual desire (Parish et al., 2021). Hypoactive sexual desire, the decrease or absence of sexual desire, is a prevalent issue in women with a multifactorial etiology, requiring both psychosocial and pharmacological interventions (Edinoff et al., 2022). Testosterone has been shown to be an effective treatment for hypoactive sexual desire in women with surgical menopause, improving sexual desire and the frequency of satisfying sexual episodes (Wheeler and Guntupalli, 2020; Uloko et al., 2022).

In addition to biological factors, various sociodemographic and psychological factors, such as age, motherhood, and sexual attitudes, influence inhibited sexual desire. Erotophilia and erotophobia represent two ends of a continuum reflecting individual attitudes toward sexual stimuli. Erotophilia involves a positive response to sexual topics, characterized by comfort, openness, and reduced anxiety. In contrast, erotophobia involves negative responses, manifesting as anxiety, discomfort, or avoidance of sexual situations (Milhausen et al., 2019; Balzarini et al., 2020). Rather than a simple dichotomy, this continuum captures the spectrum of how individuals interpret and respond to sexual stimuli. These differences have important implications for sexual health and the prevention of sexual dysfunctions, as both extremes can shape one’s sexual well-being and behaviors.

Understanding the influence of these factors, along with others such as homophobia, will allow for the development of more targeted and effective treatments for hypoactive sexual desire in women (Malary et al., 2015; Parish et al., 2021). Furthermore, the neurochemical basis of sexual desire is influenced by neurotransmitters like dopamine and norepinephrine, which play excitatory roles in the female sexual response, while serotonin has an inhibitory effect. Recent studies suggest that flibanserin can restore sexual desire by modulating these brain components that regulate the sexual response (Pfaus et al., 2022).

Various biological and psychological factors can affect a woman’s sexual desire. Oral contraceptives and anabolic androgens can cause a decrease in sexual desire because they reduce the activation of neurochemical systems in the brain that excite desire, which is stimulated by estrogens and androgens (Mernone et al., 2019). Likewise, the decrease in ovarian sex hormones and their production, as well as the consumption of medications, trauma to the pelvic region, and mental illnesses such as depression, may be other factors that contribute to the appearance of hypoactive sexual desire disorders in women (Lara et al., 2017; Johansen et al., 2020).

Furthermore, vitamin D deficiency has been linked to hypoactive sexual desire disorders in men and women. Recent studies have demonstrated a role for vitamin D in regulating sexual function, and low vitamin D levels have been associated with the development of sexual disorders (Espitia De La Hoz, 2020). In the case of pregnancy, it is important to note that this may be an important biological factor contributing to reduced sexual desire in women. During pregnancy, there can be a lack of sexual desire that increases in the third trimester and a decrease in frequency and desire throughout the pregnancy. In addition, in the third trimester, the decrease in the frequency of coital interactions can be abrupt (Jawed-Wessel and Sevick, 2017). In a study carried out in Brazil with 778 pregnant women, it was estimated that, for the most part, there was a 51% decrease in sexual desire. It has also been found that, during lactation, in the third and fourth postpartum months, there was a decrease in sexual desire due to hormonal changes (De Dios et al., 2016).

Hypoactive sexual desire disorder can be caused by sexual dysfunctions or etiological factors such as hypogonadism, hypothyroidism, functional hyperprolactinemia, pituitary tumors, autoimmune disorders, multiple sclerosis, pelvic floor dysfunction, and Klinefelter síndrome (Parish and Hahn, 2016). According to Kaplan, decreased sexual desire may be related to the inhibition of other sexual phases, although it is possible to experience arousal and orgasm without feeling full pleasure (Kaplan, 1977). In women, decreased sexual desire is more common during surgical menopause (Parish and Hahn, 2016).

Psychological difficulties, such as low self-esteem, anxiety, and depression, are important factors in the development of this disorder (De La Hoz, 2021; Figueira et al., 2021). Many women may experience a decrease in self-esteem, which could be linked to difficult experiences such as childhood sexual abuse, fear of sexuality, or marital problems (Figueira et al., 2021). Kaplan’s triphasic model indicates that psychological preparation for sex occurs during the desire phase, and each positive sexual experience reinforces this desire (Jayne et al., 2017). However, many women remain undiagnosed and untreated due to the shame or stigma associated with seeking help (O’Loughlin and Brotto, 2020).

Couple relationships are important in sexual desire, as relationship issues can increase the risk of sexual dysfunctions, especially after negative sexual experiences (Montejo et al., 2018). Negative attitudes toward sexuality further contribute to decreased desire, while sexual fantasies may stimulate it (Mark et al., 2019). Emotional support within the relationship influences the maintenance of sexual desire over time (Jones et al., 2022). However, external factors such as modern life stress, financial problems, academic pressure, or a distorted body image can also trigger or exacerbate the disorder (Niolu et al., 2016).

Cognitive-behavioral therapy has been shown to be effective in treating HSDD (ter Kuile et al., 2010). This treatment not only addresses low sexual desire but may also resolve other related sexual dysfunctions (Weibel et al., 2020). Additionally, couple therapy can be beneficial, as this disorder often affects relationship dynamics, and improving communication and emotional connection can help in treatment (Niolu et al., 2016). Personality traits, such as inhibition or introversion, also play a role in women’s sexual desire (Avasthi et al., 2017). Erotophobia and erotophilia are important constructs that correlate with sexual desire, satisfaction, and activity. Women closer to the positive erotophilia pole tend to report greater sexual desire and satisfaction, while those with high levels of sexual inhibition often experience more difficulties (Blanc et al., 2017; van Lankveld et al., 2021).

A woman’s sexual repertoire, moderate alcohol consumption, educational level, and age are some of the factors that influence the risk of presenting hypoactive sexual desire disorder (Tetik and Yalçınkaya Alkar, 2023). On the other hand, the lack of sufficient information on sexuality during childhood and adolescence, breast cancer or cardiovascular disease, and the limitation of the sexual repertoire increase the probability of presenting this sexual dysfunction. The lack of a stable partner and a low self-perception of quality of life are risk factors for female sexual dysfunction. Not practicing religion and having a job may act as protective factors in women aged 44 or older (Artiles Pérez et al., 2006). It is important to consider these factors to effectively identify and treat sexual dysfunctions in women.

Hypoactive sexual desire in women is a disorder characterized by a persistent lack of sexual thoughts, fantasies, and activity. This issue can significantly impact women’s quality of life, affecting their psychological well-being and depriving them of pleasurable sexual experiences. Hypoactive sexual desire is a multifactorial condition, influenced by medical, hormonal, psychological, and interpersonal factors. The definition of normal sexual desire in women is variable and depends on several individual and contextual factors. Testosterone and estrogen are important hormones for sexual desire, while high levels of dopamine and norepinephrine enhance it; conversely, serotonin acts as a sexual inhibitor. Identifying the factors associated with a decrease in sexual desire is critical for addressing the issue effectively. This study explores the relationship between factors such as age, motherhood, erotophobia, erotophilia, homophobia, and other sociodemographic variables with inhibited sexual desire in women from Quito, Ecuador. The findings could inform of interventions aimed at improving women’s sexual health and provide valuable insights for health professionals working in this field.

## 2 Materials and methods

### 2.1 Study design and participants

This study aimed to examine the relationship between various factors and hypoactive sexual desire in women from Quito, Ecuador. A cross-sectional correlational study was conducted, involving 421 female volunteers aged 18 to 50. Inclusion criteria required participants to be Ecuadorian, sexually active, and willing to provide informed consent.

Exclusion criteria included diagnoses of intellectual disability or severe neurological or psychiatric disorders, such as schizophrenia, acute bipolar disorder, autism spectrum disorders with significant cognitive impairments, and dementia. These conditions were excluded because they could interfere with the participants’ ability to comprehend the self-report questionnaire or might directly affect sexual desire due to neurochemical imbalances or medication use. These criteria were established to ensure the sample could reliably report their experiences, thus allowing a valid analysis of factors associated with hypoactive sexual desire in this population.

### 2.2 Instruments

Two instruments were used to assess different aspects of sexual desire in women. The first was an adaptation of the Sexual Opinion Survey (also known as the Encuesta de Opinión Sexual Revisada (EROS) in spanish) (Carpintero and Fuertes, 1994), which consists of 20 Likert-style statements with seven response options ranging from “totally agree” to “totally disagree.” EROS measures a person’s level of erotophilia-erotophobia, placing individuals on a continuum between these two attitudes. In this study, participants with higher scores were categorized as erotophilic, indicating a positive disposition toward sexual stimuli, while lower scores identified erotophobic individuals, who tend to respond negatively to sexual situations. In this study, EROS had an internal consistency index of 0.74.

The second instrument was the Inhibited Sexual Desire Scale, comprising 15 Likert-style statements with nine response options from 1 (totally false) to 9 (totally true) (Sierra et al., 2003). This scale evaluates a person’s level of sexual desire. In this study, the Inhibited Sexual Desire Scale had an internal consistency index of 0.88. This instrument was used to assess the presence of HSDD in the participants. The application of these two instruments allowed for a comprehensive assessment, differentiating between general sexual attitudes (erotophilia-erotophobia) and the specific level of inhibited sexual desire.

### 2.3 Data collection and analysis

Data collection and analysis were conducted meticulously to ensure the validity and reliability of the results. Participants were contacted through an online Google form for convenience and accessibility, and the instruments were administered after adaptation for the present study.

Spearman’s bivariate correlation analysis was used to examine the relationship between inhibited sexual desire and factors such as erotophilia-erotophobia level, age, childbearing, and age of sexual activity onset. The SPSS program was employed to guarantee the accuracy and reliability of the analysis.

### 2.4 Ethical considerations

Participants voluntarily agreed to participate in the study and provided informed consent in accordance with established ethical standards for psychological research. Data privacy and confidentiality were maintained in compliance with information protection and ethical principles. The study adhered to the 2017 ethical principles for human subject research established by the World Medical Association (AMM) and the principles of autonomy and respect for individuals as outlined in the Declaration of Helsinki. This approach safeguarded participants’ integrity and well-being throughout the study.

## 3 Results

In the study, ANOVA (Analysis of Variance) analyses were conducted to explore the potential effects of demographic variables, such as age, on the inhibited sexual desire of the participants. Inhibited sexual desire refers to a decrease or lack of sexual interest or desire. The results, as presented in Table 1, indicated that there was a significant relationship between the age of the women and their levels of inhibited sexual desire. The *F* statistics (7.13) and the associated *p*-value (< 0.001) demonstrate that this relationship was statistically significant. In other words, the findings suggest that as women get older, they are more likely to experience a decline in their sexual desire.

**Table 1:** Comparison of inhibited sexual desire scores among women according to age ranges.

	18 to 30 years		31 to 40 years		41 to 50 years		*F*	*p*
	*M*	(SD)	*M*	(SD)	*M*	(SD)		
Inhibited sexual desire	54.24	22.29	54.95	24.00	67.19	29.06	7.13	< 0.001

*Post hoc* analysis identified that women aged 41 to 50 (*p* < 0.001) 95% CI [59.48, 74.90] had higher scores on the inhibited sexual desire scale compared to women in the age ranges of 18–30 (*p* < 0.001) 95% CI [51.49, 56.98] and 31–40 years (*p* < 0.001) 95% CI [50.39, 59.51].

Table 2 illustrates the significant influence of having children on inhibited sexual desire, with an *F*-value of 13.72 and a *p*-value <0.01. It was observed that women with children reported lower levels of inhibited sexual desire than those without children. This difference could be due to the various ways motherhood impacts women’s sexuality, including hormonal changes, emotional factors, and alterations in family dynamics and responsibilities.

**Table 2:** Comparison of inhibited sexual desire scores among women with and without children.

	Without children		With children		*F*	*p*
	*M*	(SD)	*M*	(SD)		
Inhibited sexual desire	51.15	23.46	59.83	23.93	13.72	< 0.001

*Post hoc* analysis revealed that women with children (*p* < 0.01) 95% CI [56.81, 62.85] had higher scores on the inhibited sexual desire scale than women without children (*p* < 0.001) 95% CI [47.67, 54.63].

In the descriptive analysis of correlated variables (Table 3), the participants’ mean age was 30.31 years, with a standard deviation of 7.78. Concerning the variable “having children,” the mean was 0.58, with a standard deviation of 0.49. The average values for erotophobia, erotophilia, homophobia, and unconventional sex were 32.82, 21.28, 10.48, and 9.54, respectively, with standard deviations of 12.42, 7.29, 4.71, and 4.46. Finally, the mean value for inhibited sexual desire was 56.18, with a standard deviation of 24.09. These values were derived from a total sample of 421 participants.

**Table 3:** Central tendency measures of the variables: inhibited sexual desire, age, having children, age of onset of active sexual life, and level of erotophilia-erotophobia.

Variable	*N*	*M*	SD
Age	421	30.31	7.78
Have children	421	0.58	0.49
Erotophobia factor	421	32.82	12.42
Erotophilia factor	421	21.28	7.29
Homophobia factor	421	10.48	4.71
Unconventional sex factor	421	9.54	4.46

The correlation analysis revealed significant associations between inhibited sexual desire and various factors. Participants’ age exhibited a positive correlation with inhibited sexual desire, with a correlation index (*r*) of 0.16 and a significance level (*p*) < 0.001 (Table 4). This indicates that older women tended to have higher levels of inhibited sexual desire than younger women. Having children also correlated significantly with inhibited sexual desire, with an *r* of 0.18 and a *p* < 0.05, suggesting that women with children generally experienced more inhibited sexual desire than those without children. Moreover, a significant positive correlation was observed between inhibited sexual desire and the erotophobia factor, with an *r* of 0.19 and a *p* < 0.05. While erotophobia and sexual desire are conceptually related, these results empirically confirm the role of negative attitudes toward sexuality in inhibited sexual desire, particularly in this population, where individual differences may influence the expression of desire.

**Table 4:** Correlations among variables: age, having children, erotophobia factor, erotophilia factor, homophobia factor, unconventional sex, and inhibited sexual desire.

Variable	*N*	Pearson correlation	Sig. (bilateral)
Age	421	0.16**	< 0.001
Have children	421	0.18**	< 0.001
Erotophobia factor	421	0.19**	< 0.001
Erotophilia factor	421	−0.21**	< 0.001
Homophobia factor	421	−0.18**	< 0.001
Unconventional sex factor	421	−0.07	> 0.005

**Significant at the *p* < 0.01 level.

Conversely, a significant negative correlation was found between inhibited sexual desire and the erotophilia factor, with an *r* of −0.21 and a *p* < 0.05. Although erotophilia and sexual desire share theoretical links, our findings empirically demonstrate that positive attitudes toward sexuality are associated with lower levels of inhibited sexual desire. This highlights the importance of attitudes in shaping sexual behavior, which may vary across individuals. Similarly, a significant negative correlation was observed between inhibited sexual desire and the homophobia factor, with an *r* of −0.18 and a *p* < 0.05. This suggests that women with higher levels of homophobia, or negative attitudes toward homosexuality, were less likely to experience inhibited sexual desire.

## 4 Discussion

Erotophobia and erotophilia are dynamic concepts that change over time and across various situations. Factors influencing attitudes toward sex and sexuality are diverse and complex, including previous experiences, cultural values, and sexual education. Our study found significant correlations between inhibited sexual desire and both erotophobia and erotophilia factors. While erotophobia is expected to correlate with decreased sexual desire due to negative attitudes and discomfort around sexuality (Hangen and Rogge, 2022), the correlation with high erotophilia may indicate that, despite positive attitudes, other factors such as pressure to meet sexual expectations, or personal stress may impact sexual desire. Research has shown that despite their generally higher sexual interest and satisfaction, erotophilic individuals may still experience complexities in their sexual behavior, potentially engaging in risky sexual behaviors or feeling pressured by societal expectations (Lewis et al., 2006; Rye, 2023). This suggests that women with negative (high erotophobia) or very positive (high erotophilia) attitudes toward sexuality may be at a higher risk of experiencing decreased sexual desire. The importance of a balanced, healthy attitude toward sexuality is thus relevant, as erotophilia can predict sexual behaviors but is influenced by individual and relational factors such as assertiveness and relationship closeness (Hurlbert et al., 1993).

It is important to recognize that the situation of Ecuadorian women may influence their hypoactive sexual desire. In Ecuador, women face multiple challenges that can affect their sexual well-being. Sexual education in the country is limited and often focused on preventing sexually transmitted diseases and unplanned pregnancies, rather than fostering a positive attitude toward sexuality and pleasure (Castillo Nuñez et al., 2018; Ivanova et al., 2020). Additionally, cultural and religious norms can play a significant role in women’s perception of their sexuality. For example, the influence of Catholicism in Ecuadorian culture can promote conservative values that affect women’s attitudes toward sexuality, making them feel guilty or inhibited when expressing their sexual desire (Hidalgo and Dewitte, 2021).

Our study revealed that age had a significant effect on the sexual desire of Ecuadorian women, with an *F* value of 7.13 and a *p* < 0.001. This suggests that as women age, they experience a decrease in sexual desire, which aligns with previous findings. Mernone et al. (2019) documented that sexual functioning in aging women is highly dependent on psychosocial aspects related to well-being, such as optimism and relationship satisfaction, supporting the idea that emotional and psychological factors influence sexual desire.

Sexual assertiveness also impacts sexual desire, as effective communication about sexual needs and desires positively correlated with dyadic sexual desire. Couples who communicate openly about their sexuality may experience greater sexual satisfaction. In Ecuador, encouraging couples to communicate openly and honestly about their sexual needs and desires, as well as addressing any psychological or psychiatric problems, can help improve sexual satisfaction and desire in Ecuadorian women (De Meyer et al., 2014; Pozo et al., 2015).

Motherhood also emerged as a significant factor in our findings, where women with children exhibited lower levels of sexual desire, with an *F* value of 13.72 and *p* < 0.01. This supports studies that highlight how motherhood brings shifts in identity and priorities, potentially affecting sexual desire. Women often redirect their attention and energy toward their roles as mothers, and this shift can impact their sexuality, as Montemurro and Siefken (2012) found that many women feel disconnected from their sexuality for a period after having children. However, this does not necessarily equate to a total loss of desire, but rather a reorientation toward other forms of intimacy.

The analysis of variance (ANOVA) in this study revealed that other sociodemographic factors, such as age and having children, also influence women’s inhibited sexual desire. Older women and those with children are more likely to experience inhibited sexual desire. Moreover, motherhood can significantly impact a woman’s sexual desire, a finding echoed in other studies. For example, Kaplan (1977) argued that strong sexual desire before or after marriage may decrease over time, especially with the responsibilities of raising children. This finding is further supported by research from Khajehei et al. (2015), which found that almost 64% of postpartum women experienced sexual dysfunction during the first year after childbirth, with sexual dissatisfaction being the most prevalent issue. In Ecuador, women often face gender discrimination, domestic violence, and a significant wage gap (Oduro et al., 2015). These factors can generate stress, affect self-esteem, and decrease sexual desire. Moreover, Ecuadorian women usually take on most of the domestic and childcare responsibilities, which can result in exhaustion and a decrease in sexual desire (Goicolea et al., 2015).

As explained in Fuchs et al. (2021), the likelihood of experiencing sexual dysfunction increases after childbirth, affecting up to 40% of the study population. The study examined the number of children heterosexual couples had and concluded that those with more children had fewer sexual encounters and reported lower sexual satisfaction than those with fewer children. Similar results were found in Twenge et al. (2016), suggesting that having children can negatively impact the frequency and quality of sexual relations. The impact of children on sexual desire has also been noted in the study by Twenge et al. (2017), which highlights how couples today are more likely to experience a decrease in sexual encounters compared to past generations. It is important to remember that each couple is unique, and individual circumstances can affect their sex lives. These results can, however, provide valuable information for couples considering having children and wanting to be prepared for possible changes in their relationship and sex life.

Gender dynamics in the home can also significantly influence the time and energy couples have for sexual activity. Generally, women assume most household and childcare responsibilities, leaving them with less time and energy for sexual activity. Men, however, often have fewer responsibilities at home and may be more predisposed to engage in sexual activity. This dynamic can affect the frequency and quality of sexual intercourse, especially after having children. Alomair et al. (2020) observed that cultural and religious barriers, particularly in certain communities, contribute to poor sexual and reproductive health knowledge, which can negatively impact sexual desire and access to services, especially for women in low-income contexts. To find time and energy for sexual activity, parents should prioritize their sexual relationship. They can set aside specific time for intimate moments or delegate household and childcare tasks to devote more time and energy to their sexual relationship.

A lack of sexual desire can be influenced by various psychological, psychosocial, and somatic factors. Age can affect sexual desire, as older people may experience libido decrease due to hormonal changes or age-related health issues. Stressful situations and psychiatric disorders like depression and anxiety can negatively impact libido. Other influencing factors include religiosity, relationship duration, socioeconomic status, concerns about sexual functioning, personality traits, self-esteem, fears of sexuality, childhood sexual trauma, and marital problems. Furthermore, menopause is a key factor, as McCabe and Goldhammer (2012) found that sexual desire is lower among older, postmenopausal women, particularly those in longer relationships or whose partners experienced sexual dysfunction. These findings highlight the significant role of age and menopause in sexual health, particularly among women.

Moreover, the relationship between erotophilia and sexual desire is important for understanding hypoactive sexual desire. Erotophilia, defined as a positive attitude toward sexuality, can mediate how women perceive their sexual desire. van Lankveld et al. (2020) suggest that attitudes toward sexuality can moderate the relationship between sexual experiences and desire, with women who exhibit higher levels of erotophilia reporting greater sexual desire. Conversely, women with negative attitudes toward sexuality tend to experience more inhibitions. This understanding could help in developing strategies to address inhibited sexual desire.

Understanding the specific conditions affecting the sexual lives of Ecuadorian women will allow for the generation of proposals. Addressing sociocultural challenges and improving sexual education in Ecuador can be key to enhancing women’s sexual health and well-being. Moreover, by supporting open communication between partners about their sexual needs and desires, as well as addressing any psychological or psychiatric issues, we can contribute to improving sexual satisfaction and desire among Ecuadorian women.

## 5 Conclusion

This study underscores the importance of maintaining a balanced and healthy attitude toward sexuality to sustain sexual desire. The findings revealed significant correlations between inhibited sexual desire and both erotophobia and erotophilia, suggesting that women with extreme attitudes toward sexuality, whether negative or highly positive, may be more likely to experience decreased sexual desire. Furthermore, sexual assertiveness was positively associated with dyadic sexual desire, highlighting the role of open communication about sexual needs in enhancing couples’ sexual satisfaction. However, other socio-demographic factors such as age, motherhood, and cultural context also play important roles in influencing women’s sexual desire. Older women and mothers were more likely to experience inhibited sexual desire, pointing to the need for tailored interventions that address the specific challenges these groups face. In the Ecuadorian context, sociocultural factors and traditional gender roles may further compound these effects.

One limitation of this study is its focus on a specific population of women from a single city in Ecuador, which restricts the generalizability of the findings to the broader population. Ecuador is a pluricultural country, and sexual attitudes and factors influencing desire may vary significantly across regions. Additionally, the study did not include women aged 50 and older, a group that may face unique challenges related to sexual desire, particularly due to menopause and aging. Therefore, future research should include a broader demographic range, covering different regions and age groups, to offer a more comprehensive understanding of the factors influencing sexual desire in Ecuador. Longitudinal studies are needed to explore how attitudes toward sexuality evolve over time and how these changes impact sexual desire. Investigating gender dynamics, the division of labor at home, and the influence of sociocultural factors on sexual activity could also provide valuable insights. Future studies should aim to assess the sexual health of marginalized groups, including older women, to better understand the full scope of factors affecting sexual desire in diverse populations. Addressing these sociocultural barriers, promoting better sexual education, and fostering open communication within relationships could be key to improving sexual health and well-being in Ecuadorian women.

## Data Availability

The raw data supporting the conclusions of this article will be made available by the authors, without undue reservation.
